# Serum Treatment of Pneumonia

**Published:** 1919-12-13

**Authors:** 


### SERUM TREATMENT OF PNEUMONIA.

### Some Experiences and a Forecast.

Although pneumonia was known to the physicians of ancient Greece, and then, as at the present day, claimed a big death toll from the human race, the treatment of the disease has not made rapid strides and is at the moment far from being all that could be desired. When we review the average yearly mortality, independent of epidemics, we must confess the orthodox treatment does not give us very much credit. Medical statistics compiled from the Registrar-General's reports gave an average death roll of 28,755 persons in England and Wales for the four years 1900-3. The returns for the years 1918-19, the period of the recent epidemic, must have far exceeded these figures. From time to time various theories of the disease, with changing methods of therapeutics, have been put forward, but as few or no controls were used for the various forms of treatment advocated it is difficult to say whether the patient that recovered would not have done so irrespective of the treatment given. So far we have not been in the position to exhibit any one specific form of treatment which has been universally acknowledged to be scientifically based upon the pathological process of the disease. That the disease is one which calls for prompt and, if possible, a specific form of treatment we all know only too well, as the battle for life will be decided in a very few days. It is therefore a crying need that we should be able to administer to our patient a specific remedy, as in diphtheria, as soon as we have made our diagnosis. The multiplicity of micro-organisms found in the sputum and blood of pneumonic patients has led to considerable confusion, as in a mixed infection it is not easy to decide which is the predominant organism. Putting aside the questions of symbiosis, pleomorphism, variants and a filterable virus we find Fraenkel's pneumococcus and a streptococcus are the commonest forms usually met with. The possibility of some close relationship between these two organisms must also be borne in mind.

### Fraenkel's Pneumococcus.

It is now established that pneumococcal pneumonia may be caused by at least four types of pneumococci classified as types I, II, III, and IV. Types I and II are the types most commonly found in pneumonic cases and in carriers who have been in contact with pneumonia cases. Types III and IV are the commonest types found in the mouths of apparently normal individuals. Type IV was found to be frequent in the pneumonias of the natives in the Rand, South Africa.

Dochez* and Avery found that the pneumococcus during its active growth throws out into the blood a soluble substance which in severe and fatal infections appears in large quantities in the urine. This soluble substance, probably an aggressin, gives a specific precipitin reaction with the anti-pneumococcic serum corresponding to the type of pneumococcus infecting the patient. It provides, therefore, a means of rapid diagnosis of the type of pneumococcus causing the patient's pneumonia, and is also of considerable prognostic importance. Again, Dochez† refers to 65 cases of pneumonia type I. class treated with homologous serum with a mortality of 7.5 per cent., compared with 25 per cent. mortality of those treated in the ordinary way.

Also Broomfield‡ treated 11 cases of type I. with anti-pneumococcic serum and found that the serum prevented hæmic infection, and that it sterilises the blood when septicæmia has arisen.

### A Personal Experience.

During the recent so-called influenza epidemic, as a diplo-streptococcus was isolated from most of his cases, the author of this note was induced to try the effect of anti-streptococcic serum polyvalent in those cases where pneumonia was well established. The results were so encouraging that it was adopted as a routine measure to treat in a like manner all his cases diagnosed as influenza on admission to hospital. The results, more fully published in the *Lancet*, November 1, 1919, are set out below§.

Fourteen cases of epidemic pneumonia were treated with anti-streptococcic serum. Of these eleven were given the serum subcutaneously, and three intravenously. The eleven cases treated by subcutaneous injections were established cases of pneumonia on admission to hospital. The right base was involved in six cases, the left base in four, in three both lungs were involved, and one was a right apical pneumonia. Of these eleven cases two died. Of the two that died, one had a history of two previous attacks of pneumonia, and the other was a very virulent type, profoundly toxæmic, and complicated by hæmotysis, small quantities of blood being continually brought up. In neither of these cases did the serum appear to have any beneficial result. On the other hand, no bad effect was noticed. The maximum amount of serum given in any of these cases did not exceed 40 c.cm. The three cases treated by intravenous injections were admitted in a moribund condition, being profoundly toxæmic. One was maniacally delirious, and two were comatose. Although in two of these cases the serum seemed to bring the temperature down a little, there was no improvement in the general condition, and all three died. In all the fatal cases both lungs were involved. The serum used was obtained from two reputable makers and was polyvalent. There was no profuse sweating of the ordinary pneumonia crisis in those cases that recovered. The maximum amount of serum given in any of these three cases was 70 c.cm.

Sixteen cases of epidemic influenza, in the early stages

* Dochez and Avery, *Jour. Exper. Med.*, 1917, XXVI., 495.

† "Dochez Serum Treat. of Pneumococcus Infect. Musser and Kelly Prevt. Treat.," 1917, IV., 225.

‡ *Johns Hopkin's Hosp. Bull.*, 1917, XXVIII., 315.

§ Wm. Hughes. the *Lancet*, Vol. II., 1919.

**Serum Treatment of Pneumonia**—(*continued*).

of the disease, were treated with antistreptococcic serum injected subcutaneously. The initial dose given in each case was 10 c.cm., repeated in twenty-four hours if the temperature was still up. In most cases there was a fall of temperature to normal, and rapid recovery after one or two injections. In no case was more than 40 c.cm. required. Many of these cases were looked upon as potential pneumonias, the respiratory murmur being feeble or inaudible, many ronchi and rales, and well-marked crepitations over one or both bases. Epistaxis occurred in three cases, and was of a profuse type; it was checked after the first dose of serum. All these cases made uninterrupted recovery without developing any sign of pulmonary consolidation or prolonged bronchial trouble.

CONCLUSIONS.

Reviewing the past and present literature on the treatment of pneumonia the author concludes that the logical and scientific method of treating by the anti-sera method will be established before very long. The successes obtained by the anti-sera treatment of meningococcic infections should certainly lead to a hope for a similar result in pneumonia. The above is only a local experience but is suggestive. Although so far the serum treatment of pneumococcal pneumonia has been proved efficacious in infections due to type I. only, it might be advantageous to have a pooled serum which could be given prior to the result of the bacteriological examination which should be carried out in every case if possible. The procedure in every case of pneumonia would then be the administration of preliminary dose of anti-streptococcic polyvalent and a pooled anti-serum of the four pneumococci type, while the bacteriological examination is being made to identify the infecting organism. The serum might be given subcutaneously or intravenously, the condition of the case being a guide as to which method should be adopted. The possibility of anaphylaxis arising, especially if serum is given intravenously, and the importance of desensitizing the patient if this is anticipated should be borne in mind. Patients should be thoroughly questioned as to whether they ever have had serum treatment before, and also as to whether they ever get attacks of asthma.

